# Spatiotemporal dynamics of the Ebola epidemic in Guinea and implications for vaccination and disease elimination: a computational modeling analysis

**DOI:** 10.1186/s12916-016-0678-3

**Published:** 2016-09-07

**Authors:** Marco Ajelli, Stefano Merler, Laura Fumanelli, Ana Pastore y Piontti, Natalie E. Dean, Ira M. Longini, M. Elizabeth Halloran, Alessandro Vespignani

**Affiliations:** 1 Bruno Kessler Foundation, Via Sommarive 18, Trento, 38123 Italy; 2 Laboratory for the Modeling of Biological and Socio-technical Systems, Northeastern University, 360 Huntington Ave, Boston, MA 02115 USA; 3 Department of Biostatistics, University of Florida, 2004 Mowry Rd, Gainesville, FL 32611 USA; 4 Vaccine and Infectious Disease Division, Fred Hutchinson Cancer Research Center, 1100 Fairview Ave. N, Seattle, WA 98109 USA; 5 School of Public Health, University of Washington, 1959 NE Pacific Street, Seattle, WA 98195 USA; 6 Institute for Quantitative Social Sciences at Harvard University, 1737 Cambridge St, Cambridge, MA 02138 USA; 7 Institute for Scientific Interchange Foundation, Via Alassio 11/c, Turin, 10126 Italy

**Keywords:** Computational modeling, Intervention strategies, Ebola epidemiology

## Abstract

**Background:**

Among the three countries most affected by the Ebola virus disease outbreak in 2014–2015, Guinea presents an unusual spatiotemporal epidemic pattern, with several waves and a long tail in the decay of the epidemic incidence.

**Methods:**

Here, we develop a stochastic agent-based model at the level of a single household that integrates detailed data on Guinean demography, hospitals, Ebola treatment units, contact tracing, and safe burial interventions. The microsimulation-based model is used to assess the effect of each control strategy and the probability of elimination of the epidemic according to different intervention scenarios, including ring vaccination with the recombinant vesicular stomatitis virus-vectored vaccine.

**Results:**

The numerical results indicate that the dynamics of the Ebola epidemic in Guinea can be quantitatively explained by the timeline of the implemented interventions. In particular, the early availability of Ebola treatment units and the associated isolation of cases and safe burials helped to limit the number of Ebola cases experienced by Guinea. We provide quantitative evidence of a strong negative correlation between the time series of cases and the number of traced contacts. This result is confirmed by the computational model that suggests that contact tracing effort is a key determinant in the control and elimination of the disease. In data-driven microsimulations, we find that tracing at least 5–10 contacts per case is crucial in preventing epidemic resurgence during the epidemic elimination phase. The computational model is used to provide an analysis of the ring vaccination trial highlighting its potential effect on disease elimination.

**Conclusions:**

We identify contact tracing as one of the key determinants of the epidemic’s behavior in Guinea, and we show that the early availability of Ebola treatment unit beds helped to limit the number of Ebola cases in Guinea.

**Electronic supplementary material:**

The online version of this article (doi:10.1186/s12916-016-0678-3) contains supplementary material, which is available to authorized users.

## Background

Guinea was likely the starting point of the West African Ebola virus disease (EVD) epidemic in 2014–2015 [1]. The country has experienced a complicated spatio-temporal disease pattern, with several waves of increasing and decreasing weekly incidence [2]. Compared to Liberia and Sierra Leone, Guinea has a fairly developed public health system that was able to prevent the virus from exploding in major urban areas with the early availability of Ebola treatment units (ETUs) and by implementing a treatment pipeline through holding centers to limit within-hospital transmission at the very beginning of the epidemic [3]. Although Guinea has suffered considerably fewer cases than Liberia and Sierra Leone, it has struggled the most to eliminate the epidemic as evidenced by the noticeable increase in the number of cases observed several times during the second quarter of 2015 [2, 3] and continued flare-ups ongoing as of April 2016. Moreover, Guinea was the setting for the cluster randomized phase 3 trial of the recombinant vesicular stomatitis virus (rVSV)-vectored vaccine that captured many of the new cases in Guinea and whose interim assessment of efficacy was released in July 2015 [4]. All of the above elements make the temporal pattern of the EVD epidemic in Guinea markedly different from what was observed in Liberia and Sierra Leone. Comparing the epidemic behavior across different countries in the framework of a common modeling approach can help to disentangle the role of different intervention policies and identify the key determinants of the 2014–2015 EVD outbreak.

Here, we develop a data-driven stochastic agent-based model for Guinea that integrates detailed demographic information at high spatial resolution. We aim to provide a computational modeling approach to quantitatively understand the determinants of the observed disease dynamics as well as the effect of intervention and vaccination strategies. The model uses microsimulations to study the effect of control measures implemented in Guinea (such as ETUs, contact tracing, and safe burials) and to disentangle the relative contribution of key drivers of the epidemic temporal pattern. Interestingly, we contrast the results obtained for Guinea with those obtained from an analogous model for Liberia and highlight the role of the different intervention policies and healthcare systems in defining each country’s epidemic dynamics. The model is also used to generate microsimulations of ring vaccination strategies with the novel rVSV vaccine to estimate the impact of vaccination, in combination with the other containment efforts, on disease elimination.

## Methods

We use a spatially structured, stochastic agent-based model at the level of a single household that integrates detailed data on Guinean demography, hospitals and holding centers [5]. Details are in Additional file 1. Briefly, the model explicitly integrates the reported daily levels of contact tracing (CT), safe burial practices, and ETU availability, and it accounts for age-specific risk of disease and heterogeneity in transmissibility across individuals. A complete list of the data used in the model is included in Additional file 1. The parameters characterizing the transmission rate in hospitals/ETUs, households, during burial ceremonies, and in the extended family/community are calibrated by Markov chain Monte Carlo sampling applied to the likelihood of the weekly number of cases among healthcare workers (HCW) (as reported to the Guinean Ministry of Health – GMoH) and in the entire general population of Guinea (as reported in the patient database of WHO). The calibration period considers official data records up until February 25, 2015.

### Synthetic population and transmission model

Each individual in the agent-based model is explicitly simulated and has an associated epidemiological status. There is empirical evidence that Ebola transmission primarily occurs between household members and with extended family members [6, 7]; therefore, we explicitly model households and the extended family (as a network of additional households). In addition, we explicitly model transmission during funerals and in hospitals/ETUs. Characterization of EVD natural history follows the structure of previous works [8]: susceptible individuals can acquire infection after contact with an infectious individual and become exposed (and asymptomatic). At the end of the incubation period, exposed individuals become infectious (and symptomatic) and can transmit the infection to household and extended family members. Infectious individuals can either be hospitalized, die, or recover. Individuals admitted to hospitals can expose susceptible HCWs and non-Ebola patients at the same hospital, while individuals admitted to ETUs are assumed to transmit to HCWs only (although with a transmission rate one order of magnitude smaller than the hospital setting [5, 9]). Hospitalized Ebola cases may either die or recover. Individuals deceased in the general community or those who die in a hospital during the initial phase of the epidemic may also transmit the infection to household and extended family members during their funeral. Individual mobility is modeled by accounting for movements of individuals (including non-Ebola patients) seeking assistance in hospitals and ETUs, the movements of individuals taking care of Ebola patients not admitted to hospitals, and the attendance of funerals.

The Guinea synthetic population considers the specific household composition and size distributions as obtained from the Demographic and Health Survey (DHS) [10]. The population is grouped into towns and villages. Prefecture capitals are put in their exact locations and with the correct number of inhabitants [11], while all other villages are distributed within the prefecture and populated in such a way so as to match the total number of inhabitants of the prefecture [11]. The number of hospitals for each prefecture was obtained from the 2007 Guinean health statistics [12]; the number of beds and HCWs for each hospital match available health statistics for Guinea from WHO [13]. In addition to hospitals, ETUs with location, opening date, number of beds, and HCWs matching the available information, are considered. A full description of the synthetic population, transmission model, and data used is available in Additional file 1.

### Non-pharmaceutical interventions

The model accounts for three non-pharmaceutical interventions: (1) ETUs, (2) community safe burials, and (3) contact tracing (CT).

1. The modeling of cases in ETUs/hospitals is as follows. Symptomatic individuals seeking care are admitted to an ETU with available beds in the same prefecture. If no ETU is available they are held for 1 day in the hospital closest to the place of residence. After 1 day in the hospital, if there are beds available in ETUs, individuals are assigned to the closest ETU having available beds; otherwise, they remain in the hospital. Additionally, each individual who is hospitalized (either in a hospital or in an ETU) has a probability to be considered as an index case for a CT investigation. Finally, individuals admitted to ETUs who die are always buried safely.
2. The GMoH reports the daily number of total burials and of safe burials in the community [3]. For each day *t* of the simulation, every individual deceased at that time in the community (i.e., who was not hospitalized) has a probability *B*(*t*) of being buried safely, thus preventing transmission events during the funeral. We compute the daily probability *B*(*t*) of being safely buried in the community as the ratio between safe and total burials (both in the community).
3. The GMoH reports the daily number of contacts followed in CT investigations [3]. We compute a proxy for the number of new contacts followed per case at day *t*, *F*(*t*), as the ratio between the total number of followed contacts over the period (*t, t* + 21) and the total number of cases over the same period. Ajelli et al. [7] estimated that the probability of identifying a case through CT depends linearly on the number of contacts followed; for instance, 11.5 traced contacts per case corresponds to about 30 % detection probability. We use this information to derive the daily probability of following a contact, Φ(*t*), from *F*(*t*). At each time step, for each individual admitted either to a hospital or an ETU, we sample from a Bernoulli distribution with probability Φ(*t*) to determine whether their household is followed or not. The same procedure is applied to each additional household in their extended family network. Once an individual belonging to a household followed by CT becomes symptomatic, he is hospitalized on the same day of symptom onset (provided that beds are available). Additional details on modeled interventions and a discussion of the underlying modeling assumptions are provided in Additional file 1.

### Age-dependent risk of infection and heterogeneity

From data reported to WHO, the age-specific incidence of Ebola is notably higher among adults versus children in Guinea, Liberia, and Sierra Leone [7, 14–16]. Therefore, the model allows children aged 0–14 years to have a different risk of infection compared to individuals aged 15 years or older [17]. By analyzing the number of cases by age, as reported by the GMoH, with a simple compartmental transmission model (Additional file 1), we found that the risk of infection of children aged 0–14 years is 0.246 (95 % CI, 0.212–0.284) times that of adults. A second important feature of EVD, similar to other diseases [18], is a high level of heterogeneity in individual transmissibility, with a few cases leading to a majority of secondary cases [6, 7, 19]. The model accounts for this feature by assuming each individual has a specific infectivity sampled from a Gamma distribution such that the overall distribution of secondary cases follows a negative binomial distribution with dispersion parameter 0.20 (95 % CI, 0.13–0.31), as estimated by analyzing the data reported in Faye et al. [6] (Additional file 1).

### EVD parameters

The majority of parameters regulating EVD natural history used in the model were taken from the study of the outbreak by the WHO Ebola response team [14] and are reported in Additional file 1. The parameters regulating transmission rates in hospitals/ETUs, in households, during burial ceremonies, and the scaling factor accounting for weaker contacts in the extended family compared to those within the household are estimated by using a Markov chain Monte Carlo approach exploring the likelihood of the recorded number of cases in the general population and among HCWs (details in Additional file 1). The dataset used for model calibration in this study is from a time period of intensive ongoing interventions. Each of the interventions mainly acts in reducing transmission in one specific setting: ETUs reduce transmission in healthcare settings; community safe burials reduce transmission at funerals; and CT reduces transmission in households and extended family. This, together with the use of two different case count curves, leads to better identifiability of transmission rates. Simulations were initialized with 52 infected individuals geographically distributed in four prefectures (namely Guéckédou, Conakry, Macenta and Dabola) based on information on the first confirmed cases reported to the WHO. The national-level simulations were run until there were 495 cases, at which point the simulated date is set to August 4, 2014, according to GMoH reports [3].

### Ring vaccination

To quantify the effect of the novel rVSV vaccine, we have simulated a ring vaccination protocol similar to that used in the Ebola ça suffit randomized trial [20]. Beginning March 23, 2015, individuals admitted to ETUs are considered as index cases for ring vaccination. Once an index case for a new ring is identified, a socio-geographical ring is defined, including contacts and contacts of contacts of the index case and individuals living in a radius of 30 meters around the index case’s house (note that the Ebola ça suffit trial included contacts and contacts of contacts only). As the ring index case is admitted to an ETU, the ring is randomized, with probability 50 %, to receive immediate vaccination (3 days after the admission of the ring index case, reflecting logistical constraints experienced in the actual trial [20]) or delayed vaccination (21 days after enrollment). Ring members are eligible for vaccination if aged 18 years or more. We assume that the vaccine is administered to 90 % of eligible members and that there is a delay of 6 days between vaccine administration and maximal protective efficacy in individuals for whom the vaccination is efficacious [4]. Three levels of vaccine efficacy are evaluated: 75 %, 90 %, or 100 % of vaccinated individuals are completely protected [4].

## Results

**Fig. 1:**
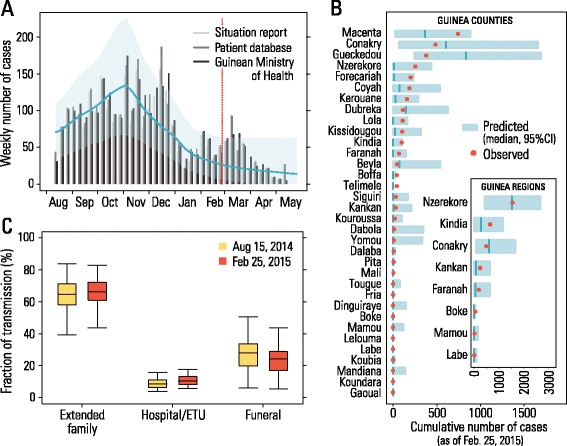
Model validation and estimates. **a** Weekly number of cases over the period August 2014 – May 2015 according to the WHO Ebola situation report, patient database and Guinean Ministry of Health (bars) and predicted by the model (the blue line is the average, and the shaded blue region is the 95 % confidence interval of simulated epidemics). The red line, set on February 25, 2015, marks the end of the calibration period. **b** Predicted and observed cumulative number of cases by prefecture and region as of February 25, 2015. **c** Boxplot of the proportions of transmission by setting as of August 15, 2014, and February 25, 2015, in order to show the variation of these quantities. August 15, 2015, is chosen in such a way as to allow a comparison with the results for Liberia presented previously [5]; February 25, 2015, corresponds to the end of the calibration period

The adequacy of the model can be analyzed by comparing the official data through mid-May 2015 with the model output according to the posterior distributions of the parameters. The weekly incidence produced by the model in shows a temporal pattern similar to the observed data and captures the slow decay in incidence beginning in March 2015 (see Fig. 1a). The two data points in December 2014 that fall just outside the 95 % CI of model predictions are likely linked to superspreading events that occurred during two traditional (unsafe) burials reported in Kissidougou [1–3, 21]. A similar temporal pattern of case counts is estimated by the model when assuming that only 80 % of cases have been reported to WHO (Additional file 1). Although model calibration considers only nationally aggregated data, we can check the model adequacy by comparing model results at a finer geographical resolution with the available data (Fig. 1b). Both the data and model output show that the most affected regions are Nzérékoré (in particular Macenta and Guéckédou prefectures), the region where EVD was first seeded; Conakry, the capital region with an operational ETU since March 2014; and Kindia, a region close to Conakry (in particular Coyah, Dubréka and Forécariah prefectures). Further analyses including the temporal pattern at the prefecture level and cumulative attack rates by region are reported in Additional file 1. These results suggest that, although the calibration does not contain explicit information on intervention timing and intensity for each specific region, the dynamics of the model adequately reproduce the spatial heterogeneity observed in the epidemic [6].

**Fig. 2:**
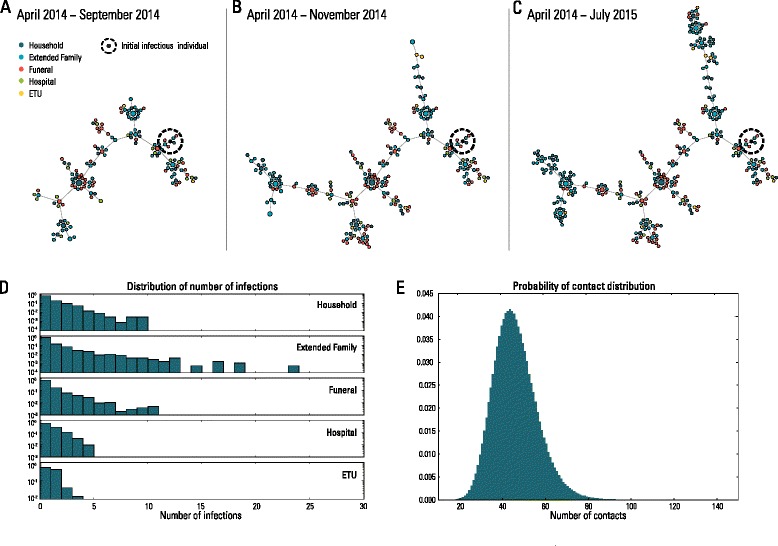
Transmission tree and distribution of secondary infections. **a** Transmission tree of one randomly chosen epidemic obtained by simulating the calibrated model. Different colors represent the setting where the individual was infected. As the whole transmission tree would have been too wide to display on a page (thousands of edges on average), we show only one component. Simulated infections occurring over the period April 2014 to September 2014 are shown. **b** As **a**, but showing infections over the period April 2014 to November 2014. **c** As **a**, but showing infections over the period April 2014 to July 2015 (i.e., the entire simulated epidemic). **d** Distribution of the number of secondary cases by setting as obtained by the analysis of the whole transmission tree reported in panel **c**. **e** Degree distribution of contacts with members of the same household and of the extended family as resulting from the model

The estimated proportions of EVD transmission attributable to each of the three modeled transmission settings are shown in Fig. 1c. Using data through February 25, 2015, modeling results suggest that 65.7 % (95 % CI, 43.7–82.8) of cases were due to transmission between household and extended family members, 23.4 % (95 % CI, 5.3–43.6) were caused by unsafe burials, and only 10.9 % (95 % CI, 5.7–17.7) occurred in hospitals and ETUs. The distribution of secondary infections by setting is highly variable due to different numbers of potential contacts across different settings and individual variation in infectivity. Specifically, as individuals are explicitly considered in the model, we are able to identify the infector of each case, and thus to reconstruct the whole transmission tree for each simulation. The simulated transmission trees (an example is reported in Fig. 2a, b, c) show high heterogeneity in the number of secondary infections, with few individuals responsible for the majority of infections. This result is in good agreement with reports from other studies (see for instance [6, 7, 19]). Moreover, we found that the distributions of secondary cases differ by setting, although they are all highly skewed (Fig. 2d). Interestingly, we found highly skewed distributions, to the right, of secondary cases in the household and extended families, although the distribution of contacts between individuals in the extended family network shows a well behaved distribution of about 44 contacts on average (Fig. 2e).

**Fig. 3:**
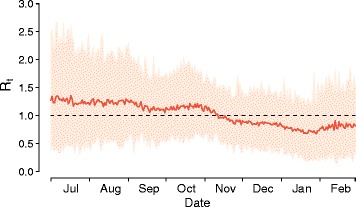
Reproductive number over time. *Rt* as computed from the time series of cases obtained by simulating the calibrated model (details on the computation of *Rt* are reported in Additional file 1). Mean and 95 % credible interval are shown

The simulated case series generated by the model allows the reproductive number *R*_*t*_ to be estimated as a function of time (details in Additional file 1). We estimated the mean *R*_*t*_ in early July 2014 to be 1.27 (95 % CI, 0.36–2.49). In early November 2014, *R*_*t*_ decreased below the critical threshold (Fig. 3). This suggests sustained exponential growth in the very early phases of the epidemic. Using a simple compartmental model to analyze age-specific incidence data early in the epidemic, not accounting for intervention strategies (Additional file 1), we found a consistent estimate of the reproduction number R. Specifically, we estimated the mean reproduction number to be 1.18 (95 % CI, 1.17–1.19). Estimates obtained by using both approaches are remarkably lower than estimates for neighboring countries [5, 22, 23].

**Fig. 4:**
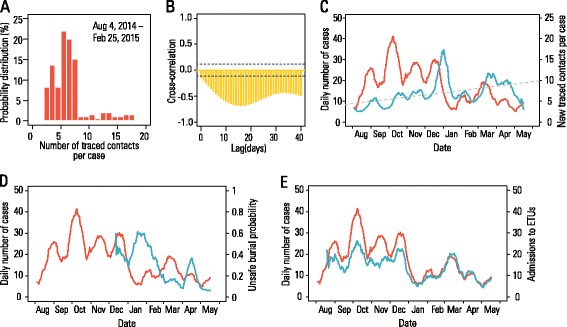
Correlations between interventions and number of cases. **a** Probability distribution of number of traced contacts per case over the period August 4, 2014, to February 25, 2015. **b** Cross-correlation between the average number of contacts included in contact tracing per case at a given time and the incidence of cases observed 0 to 40 days later. The average number of contacts included in contact tracing per case is computed as the sum of followed contacts over 21 days divided by the sum of new cases over the same 21 days (alternative definitions of contact tracing are considered in Additional file 1). The highest absolute value of cross-correlation is obtained for a lag of 17 days. **c** Red line: daily number of cases (as obtained with a moving average of 15 days, i.e., 1 week previous and 1 week following the data point) over time; blue line: number of traced contacts per case (defined as in **b**) over time; dotted line: linear model best fitting the number of traced contacts. Scale for blue and dotted curves is on the right axis. **d** Red line: as in **c**; blue line: probability of unsafe burials over time computed as the fraction of daily community safe burials over the daily total number of community burials (scale on the right axis); the curve is then obtained by computing a moving average of 15 days, i.e., 1 week previous and 1 week following the data point. **e** Red line: as in **c**; blue line: number of admissions to ETUs over time (scale on the right axis); the curve is then obtained by computing a moving average of 15 days, i.e., 1 week previous and 1 week following the data point. Dates in panels **c**–**e** refer to the period August 2014 to June 2015

The overall behavior of epidemic incidence over time can be readily linked to temporal variation in the level of CT. A precise quantification of the key role of CT (whose variability is shown in Fig. 4a) is provided by the time series correlation analysis of epidemic incidence with intervention indicators such as CT, safe burials, and ETU admissions. We find that the average number of contacts per case included in CT at any given time is strongly negatively correlated with the incidence of cases observed 10–30 days later (Fig. 4b). The correlation is most negative at a lag of 17 days (*ρ* = *−*0*.*74, *P <* 0*.*001), meaning that CT effort at a given time has its greatest effect on the number of cases that will be observed 17 days later. Fig. 4c shows that (1) the average level of CT has increased over time (the linear model best fitting the data shows an average increase of 0.53 persons per month, *P <* 0*.*001); and that (2) increasing CT is associated with a decrease in incidence after a delay. The impact of safe burial procedures has been rather constant during the course of the epidemic. Indeed, the number of unsafe burials itself has remained quite constant (Fig. 4d; no significant correlation was found at any lag between 0 and 30 days; Additional file 1). As expected, we find a positive correlation (Fig. 4e) between the number of admissions to ETUs and daily incidence (*ρ* = 0.91, *P <* 0*.*001, without lag; Additional file 1). Based on a partial correlation analysis, we found that the significant negative correlation between observed cases and CT still holds after correcting for the effect of ETU admission (Spearman partial correlation coefficient: −0.295, *P <* 0*.*001). We estimate that the average probability of hospitalization (approximated crudely as the number of cases admitted to ETUs divided by the total number of cases over the considered time) has increased over time – average increase: 4.3 % per month, *P <* 0*.*001; mean over the entire period: approximately 83 %. This result is adequately reproduced by the model driven by the CT data. The low level of CT observed in August–October 2014 corresponds to an increase in the number of weekly cases until early November 2014 (Fig. 1a). Following higher levels of CT observed in November, the number of cases in the model sharply decreases until the beginning of January 2015 (from about 130 to about 50 weekly cases). In February 2015, the intensity of CT was reduced, leading to a slower decline in disease incidence with potentially new epidemic waves, as shown by the observed peak of cases recorded in both WHO and GMoH data in mid-March 2015.

**Fig. 5:**
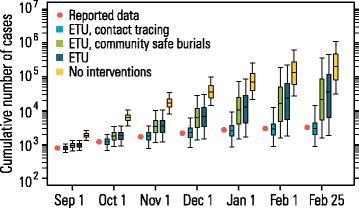
Disentangling the impact of different interventions. Boxplots for the cumulative number of cases from September 2014 through February 2015 assuming different interventions

The model allows us to disentangle the effects of different interventions in Guinea by analyzing counterfactual scenarios in which specific interventions are not implemented (Fig. 5). In the absence of any intervention, the estimated median number of cases by February 25, 2015, would have been 204,225 (95 % CI, 31,597–1,090,032), corresponding to an attack rate of 1.9 % (95 % CI, 0.3–10.3). With case isolation in ETUs and safe burials, the median number of cases decreases to 21,263 (95 % CI, 1,587–360,832; i.e., 0.2 % attack rate, 95 % CI, 0.01–3.4), and decreases further to 3,002 (95 % CI, 869–14,287; i.e., 0.03 % attack rate, 95 % CI, 0.001–0.1) with case isolation in ETUs and CT, in line with observed data. These results confirm the key role of ETUs and CT for controlling the epidemic and suggest that traditional burials, while possibly leading to superspreading events that are most relevant when the prevalence is low, are not the main driver of the epidemic.

**Fig. 6:**
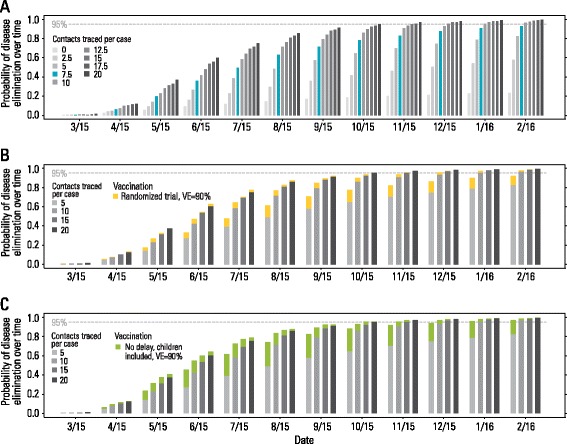
Impact of interventions on disease elimination. **a** Probability of disease elimination over time, assuming different values for the number of traced contacts per case since February 25, 2014. Blue bars represent a situation comparable to what was observed in April 2015. **b** As **a** but assuming ring vaccination starting on March 23, 2015, enrollment 3 days after the admission of ring index cases to ETU, 90 % vaccine efficacy, 6 days for vaccinated individuals to develop protective immunity, and vaccine administered to 90 % of adults (≥18 years old). In 50 % of rings, vaccine is administered with a delay of 21 days with respect to immediately vaccinated rings. **c** As **b** but vaccine is administered to 90 % of all individuals and all rings are vaccinated at the time of enrollment. The number of traced contacts matches the data (see Methods and Additional file 1) until February 25, 2015; then, it is assumed to be constant over time until the end of the simulation, at the level reported in the legend

These results suggest that the level of CT is a key parameter for controlling an Ebola epidemic. Since CT has fluctuated considerably over time, we provide different scenarios that project the probability of disease elimination in Guinea assuming variable levels of CT (after February 25, 2015). Model simulations highlight that both the probability of disease elimination and the number of secondary cases vary remarkably on the basis of CT levels (Fig. 6 and Additional file 1). If 20 contacts are traced per Ebola case, the estimated probability of disease elimination by February 2016 is 99.5 % (the estimated cumulative number of cases would be 4845 at most). In contrast, if no CT is performed, disease elimination becomes unlikely (23.6 % probability).

An interim assessment reported an estimated 100 % (95 % CI, 74.7–100) vaccine efficacy [4], opening the path to vaccination strategies aimed at EVD elimination. The agent-based model presented here allows us to simulate the ring vaccination strategy. In particular, we assume that the ring vaccination strategy, roughly corresponding to the protocol, started on March 23, 2015, and we estimate the added impact of ring vaccination on disease elimination. In Fig. 6b we report the increase in the probability of disease elimination due to ring vaccination at different points in time, assuming a vaccine efficacy of 90 % and with different levels of CT. The major benefit of ring vaccination is observed at low levels of CT (5–10 individuals per case). In this range of CT, the probability of disease elimination increases noticeably by performing a ring vaccination strategy if individuals of all ages are vaccinated without delays (Fig. 6c). In Additional file 1, we report a sensitivity analysis for varying vaccine efficacy. Briefly, this analysis shows that the impact of ring vaccination on EVD elimination when assuming low vaccine efficacy (i.e., 75 %) is remarkable only at low levels of CT.

## Discussion

The modeling approach presented here adequately captures the complex spatiotemporal pattern of the Guinea EVD epidemic through microsimulations. This computational modeling approach was previously applied to the EVD epidemic in Liberia, allowing us to compare the results obtained for the two countries to determine how differences in healthcare infrastructure and the implementation of interventions can explain the distinct epidemic patterns. For Guinea, the model yields an estimated reproduction number (*R ~* 1*.*2) that is much lower than those reported for Liberia (estimates in the range 1.7–2.0) [5, 14, 22, 23] and Sierra Leone (estimates in the range of 1.4–2.0) [14, 23, 24]. The model suggests that this particular feature of the epidemic in Guinea stems from the early availability of ETUs (which were already open in the phase where we computed the reproduction number) and their more developed healthcare system that helped to prevent sustained exponential growth of the epidemic. This seems to be consistent with evidence from the field that a lower proportion of cases in Guinea were associated with transmission in healthcare settings compared with Liberia; in particular, the analysis of the initial transmission chain in Conakry attributed 9 % of EVD infections to hospitals over the period April to August, 2014 [6]. Consistently, we estimate that 8.8 % (95 % CI, 3.6–15.8) of transmissions were linked to hospital/ETU contacts through August 15, 2014. A similar proportion of transmissions in hospital settings (namely 7.7 %) was reported in an analysis of an infection tree in Pujehun district of Sierra Leone [7]. On the other hand, these estimates differ substantially from analogous estimates in Liberia where, according to modeling results [5], a much larger proportion of transmission (30.8 %) was attributed to hospitals, especially in the initial phase of the epidemic. This difference is likely due to the lack of sufficient beds in ETUs in Liberia in the initial phase of the epidemic. In Guinea, even at the time of the peak incidence (November–December 2014), the daily number of admissions to ETUs ranged from about 10 to 30, lower than the estimated bed capacity of the four ETUs already open at that time. The estimated proportion of transmission linked to unsafe burials is higher than that previously observed [6] over the period February 10 to August 25, 2014. However, that analysis preceded our current one and was not able to take the subsequent rise in the number of unsafe burials into account (Fig. 4d). Our analysis shows the key role of CT for limiting Ebola spread. An analysis of the Ebola epidemic in Liberia [5] showed that the distribution of household protection kits had the effect of significantly accelerating the elimination phase. In sum, the results of both studies highlight the importance of reducing transmission between household and extended family members for controlling Ebola spread.

The model also indicates that the long tail of the disease elimination phase in Guinea could be explained by fluctuations in the level of CT that, although adequate to prevent resurgence of a large epidemic [25], were not enough for timely elimination of the disease (Fig. 6). Specifically, we found numerical evidence that 7–10 contacts traced per Ebola case were consistently needed to achieve disease elimination with relatively high probability (*>*90 %) before the end of 2015. To effectively perform CT, human and logistical resources are required. Human behavior, such as fear and hostility, can also drive acceptance of CT, as well as the fraction of population seeking care in ETUs and safe burials for the deceased [26]. Thus, the estimated impact of the interventions that we report incorporates population compliance to public health directions; different human behavioral responses of the population would lead to increased or decreased effectiveness of control measures. Indeed, this represents a possible explanation for the observed larger effect of CT in 2015 than in 2014 (Fig. 4c). Contact tracers could have become more skilled over the course of the epidemic after acquiring experience on the ground. Alternatively, the population could have changed its behavior, allowing health personnel to perform CT more efficiently. However, our modeling analysis does not have the capability to disentangle these effects.

Another important element to consider in discussing disease elimination scenarios is the WHO-sponsored ring vaccination trial of the rVSV vaccine in Guinea. The interim data analysis shows an impressive 100 % vaccine efficacy [4]. Numerical simulations show that it is reasonable to assume that the vaccination trial itself may have had a considerable impact on the disease control effort. These simulations show that the vaccination program provides the largest benefit in regions with low, deteriorating or fluctuating CT, thereby considerably accelerating disease elimination. Consistent with other modeling studies [27–29], we found that the major benefit of ring vaccination is observed at low levels of CT (5–10 individuals per case). Finally, vaccination gives direct protection to vaccinees, which is important for controlling epidemics on a local level as well as protecting those providing front line care.

Most of the assumptions and limitations of the model have already been discussed by Merler et al. [5]. It is worth mentioning however that, similar to the model for Liberia, we assume that Ebola cases in Guinea are unlikely to travel long distances when they have active symptoms, unless they are seeking hospital care or help from relatives and friends. This assumption has been validated for Liberia and appears to adequately reproduce the geographical incidence in Guinea. However, we cannot exclude the possibility that local population mobility could drive EVD dynamics, especially during the elimination stage where the epidemic can be dominated by rare fluctuations. Another feature that we have not considered in the model is the effect of asymptomatic infection and acquired immunity [30]. While the early stage of the epidemic is probably not overly affected by asymptomatic infections, acquired immunity may play a role in speeding up the elimination of the disease. Notwithstanding the mentioned limitations, the model is able to adequately describe the dynamics of the epidemic in Guinea, provide estimates of transmission by setting in agreement with reported data [6], and capture the highly heterogeneous pattern of transmission reported in previous studies [6, 7]. These findings support our choice of using an individual-based modeling approach, capturing the observed high heterogeneity in the number of secondary infections and the clustering effect due to the low number of effective contacts in the different settings.

## Conclusions

Our findings demonstrate the usefulness of computational modeling approaches in which explicit assumptions can be validated across epidemic outbreaks in different countries. Contrasting the results obtained for Guinea and Liberia [5], it is possible to characterize the role of interventions in both countries and their effect on the spatiotemporal pattern of the outbreak. In particular, our analysis identifies contact tracing, together with the early availability of beds in Ebola treatment units, as key drivers of the different patterns of spread observed in Guinea, Liberia, and Sierra Leone. This study lends confidence to the assumptions and modeling choices used and makes a strong case for extending the model to Sierra Leone, and to the analysis of vaccination trials and campaigns. Finally, it provides a computational approach for the preparation and analysis of contingency plans, including vaccination strategies, for future EVD outbreaks in other countries.

## Additional file

Additional file 1: Supplementary information. Text describing methods in detail and additional results. (PDF 3870 kb)

## Abbreviations

CT: Contact tracing
ETU: Ebola treatment unit
HCW: Health care worker
EVD: Ebola virus disease
GMoH: Guinean ministry of health
CI: Confidence interval

## References

[1] World Health Organization. WHO Global Alert and Response. One year into the Ebola epidemic: a deadly, tenacious and unforgiving virus. 2015. http://www.who.int/csr/disease/ebola/one-year-report/introduction/en/. Accessed 29 Aug 2016.

[2] World Health Organization. WHO Global Alert and Response. Situation reports: Ebola response roadmap (2014–2015). http://apps.who.int/ebola/ebola-situation-reports. Accessed 29 Aug 2016.

[3] Guinean Ministry of Health. Rapport de la Situation Epidemiologique Maladie a Virus Ebola en Guinee. 2014–2015. In French. http://guinea-ebov.github.io/sitreps.html. Accessed 29 Aug 2016.

[4] Henao-Restrepo AM, Longini IM, Egger M, Dean NE, Edmunds WJ, Camacho A, Carroll MW, Doumbia M, Draguez B, Duraffour S, Enwere G, Grais R, Gunther S, Hossmann S, Konde MK, Kone S, Kuisma E, Levine MM, Mandal S, Norheim G, Riveros X, Soumah A, Trelle S, Vicari AS, Watson CH, Keta S, Kieny MP, Rottingen J-A (2015). Efficacy and effectiveness of an rVSV-vectored vaccine expressing Ebola surface glycoprotein: interim results from the Guinea ring vaccination cluster-randomised trial. Lancet.

[5] Merler S, Ajelli M, Fumanelli L, Gomes MF, Pastore y Piontti A, Rossi L, Chao DL, Longini IM, Halloran ME, Vespignani A (2015). Spatiotemporal spread of the 2014 outbreak of Ebola virus disease in Liberia and the effectiveness of non-pharmaceutical interventions: a computational modelling analysis. Lancet Infect Dis.

[6] Faye O, Boelle P-Y, Heleze E, Faye O, Loucoubar C, Magassouba N, Soropogui B, Keita S, Gakou T, Koivogui L (2015). Chains of transmission and control of Ebola virus disease in Conakry, Guinea, in 2014: an observational study. Lancet Infect Dis.

[7] Ajelli M, Parlamento S, Bome D, Kebbi A, Atzori A, Frasson C, Putoto G, Carraro D, Merler S (2015). The 2014 Ebola virus disease outbreak in Pujehun, Sierra Leone: epidemiology and impact of interventions. BMC Med.

[8] Legrand J, Grais RF, Boelle P-Y, Valleron A-J, Flahault A (2007). Understanding the dynamics of Ebola epidemics. Epidemiol Infect.

[9] Meltzer MI, Atkins CY, Santibanez S, Knust B, Petersen BW, Ervin ED, Nichol ST, Damon IK, Washington ML (2014). Estimating the future number of cases in the Ebola epidemic – Liberia and Sierra Leone, 2014–2015. Morb Mortal Wkly Rep.

[10] The DHS Program. Guinea Demographic and Health Survey 2005. Final Report. 2005. http://dhsprogram.com/publications/publication-FR162-DHS-Final-Reports.cfm. Accessed 29 Aug 2016.

[11] Institut National de la Statistique Guinee. Resultats definitifs du Troisieme Recensement General et de la Population et de l'Habitation. (In French) 2014. http://www.stat-guinee.org/index.php/res-def-rgph3. Accessed 29 Aug 2016.

[12] Institut National de la Statistique Guinee: Guinee - Annuaire des statistiques sanitaires 2007. 2007. [In French] http://www.stat-guinee.org/nada/index.php/catalog/1. Accessed 29 Aug 2016.

[13] World Health Organization. WHO Regional Office for Africa. Country Health Profile – Guinea Factsheets of Health Statistics 2010. 2010. http://www.aho.afro.who.int/profiles_information/index.php/Guinea:Index?lang=en. Accessed 29 Aug 2016.

[14] WHO Ebola Response Team (2014). Ebola virus disease in West Africa: the first 9 months of the epidemic and forward projections. N Eng J Med.

[15] WHO Ebola Response Team (2015). West African Ebola epidemic after one year: slowing but not yet under control. N Engl J Med.

[16] WHO Ebola Response Team (2015). Ebola virus disease among children in West Africa. N Engl J Med.

[17] Dowell SF (1996). Ebola hemorrhagic fever: why were children spared?. Pediatr Infect Dis J.

[18] Lloyd-Smith JO, Schreiber SJ, Kopp PE, Getz WM (2005). Superspreading and the effect of individual variation on disease emergence. Nature.

[19] Althaus CL (2015). Ebola superspreading. Lancet Infect Dis.

[20] Ebola ça suffit ring vaccination trial consortium (2015). The ring vaccination trial: a novel cluster randomised controlled trial design to evaluate vaccine efficacy and effectiveness during outbreaks with special reference to Ebola. BMJ.

[21] Victory K, Coronado F, Ifono S, Soropogui T, Dahl B (2015). Ebola transmission linked to a single traditional funeral ceremony – Kissidougou, Guinea, December, 2014-January 2015. MMWR Morb Mortal Wkly Rep.

[22] Chowell G, Nishiura H (2014). Transmission dynamics and control of Ebola virus disease (EVD): a review. BMC Med.

[23] Nishiura H, Chowell G (2014). Early transmission dynamics of Ebola virus disease (EVD), West Africa, March to August 2014. Euro Surveill.

[24] Kucharski AJ, Camacho A, Checchi F, Waldman RJ, Grais RF, Cabrol J-C, Briand S, Baguelin M, Flasche S, Funk S, Edmunds WJ (2015). Evaluation of the benefits and risks of introducing Ebola community care centers, Sierra Leone. Emerg Infect Dis.

[25] Browne C, Gulbudak H, Webb G (2015). Modeling contact tracing in outbreaks with application to Ebola. J Theor Biol.

[26] Chan M (2014). Ebola virus disease in West Africa – no early end to the outbreak. N Eng J Med.

[27] Wells C, Yamin D, Ndeffo-Mbah ML, Wenzel N, Gaffney SG, Townsend JP, Meyers LA, Fallah M, Nyenswah TG, Altice FL (2015). Harnessing case isolation and ring vaccination to control Ebola. PLoS Neglect Trop Dis.

[28] Kucharski AJ, Camacho A, Flasche S, Glover RE, Edmunds WJ, Funk S (2015). Measuring the impact of Ebola control measures in Sierra Leone. Proc Natl Acad Sci.

[29] Kucharski AJ, Eggo RM, Watson CH, Camacho A, Funk S, Edmunds WJ (2016). Effectiveness of ring vaccination as control strategy for Ebola virus disease. Emerg Infect Dis.

[30] Bellan SE, Pulliam JRC, Dushoff J, Meyers LA (2014). Ebola control: effect of asymptomatic infection and acquired immunity. Lancet.

