# Supplementary material for: Spatiotemporal dynamics of the Ebola epidemic in Guinea and implications for vaccination and disease elimination: a computational modeling analysis
Source: BMC Med. 2016 Sep 7;14(1):130. doi: 10.1186/s12916-016-0678-3 (PMC5013652; doi:10.1186/s12916-016-0678-3)
Supplement: Additional file 1: — Supplementary information. Text describing methods in detail and additional results. (PDF 3870 kb) [file 12916_2016_678_MOESM1_ESM.pdf]

Spatio-temporal dynamics of the Ebola epidemic in Guinea and implications for vaccination and disease elimination: a computational modeling analysis

Marco Ajelli[1], Stefano Merler[1], Laura Fumanelli[1],
Ana Pastore y Piontti[2], Natalie E. Dean[3],
Ira M. Longini Jr.[3], M. Elizabeth Halloran[4,5], Alessandro Vespignani[2,6,7]

[1]Bruno Kessler Foundation, Trento, Italy
[2]Laboratory for the Modeling of Biological and Socio-technical Systems,
Northeastern University, Boston, MA 02115, USA
[3]Department of Biostatistics, College of Public Health
and Health Professions, and Emerging Pathogens Institute,
University of Florida, Gainesville, FL 32611, USA
[4]Vaccine and Infectious Disease Division,
Fred Hutchinson Cancer Research Center, Seattle, WA 98109, USA
[5]Department of Biostatistics, University of Washington, Seattle, WA 98195, USA
[6]Institute for Quantitative Social Sciences
at Harvard University, Cambridge, MA 02138, USA
[7]Institute for Scientific Interchange, Torino, Italy

[*]corresponding author; E-mail: a.vespignani@neu.edu.

Supplementary Information

Contents

1 Data on Guinea sociodemographic structure and EVD interventions **3**

2 Transmission model and Ebola natural history **4**
2.1 Transmission within households 5
2.2 Transmission in the extended family 5
2.3 Transmission during burial ceremonies 6
2.4 Transmission in hospitals and ETUs 6
2.5 Overall force of infection and probability of being infected 7
2.6 Modeling non-pharmaceutical intervention measures 7
2.6.1 ETU 7
2.6.2 Community safe burial 8
2.6.3 Contact tracing investigation 8

3 Markov chain Monte Carlo calibration **9**

4 Age-specific risk of infection **12**

5 Heterogeneous transmissibility **14**

6 Reproduction number over time **15**

7 Geographical spread **15**

8 Analysis of performed intervention measures **17**

9 Probability of disease elimination **18**

Bibliography **19**

1 Data on Guinea sociodemographic structure and EVD interventions

According to the latest census data [1], Guinea has a population of 10,628,972 inhabitants residing in an area of 245,836 km^2, and subdivided into 8 regions and 34 prefectures. In our model, we placed the capital of each prefecture in the exact location given by GPS coordinates and with the exact number of inhabitants as obtained from available data. For the remainder of the territory of the prefecture, we generated villages of about 400 inhabitants on average, randomly located over the territory, until the total population of the prefecture was obtained. By using a standard procedure [2, 3], modeled individuals were grouped into randomly assigned households built in such a way that household size distribution and age structure within households match data available from the Demographic and Health Surveys (DHS) Program [4] (see Fig S1).

According to the 2007 Guinean health statistics [5], there were 44 hospitals in Guinea, of which the prefecture of reference is known; at least one hospital was present in each prefecture. Number of beds and health care workers match available health statistics obtained from the WHO [6]. In our model, all hospitals were always open, even in conjunction with the opening of ETUs, and worked at full capacity. In addition to hospitals, Ebola treatment units (ETUs) were considered in the model, each operating in the exact prefecture and since the exact date. Simulated number of beds and health care workers for these facilities also match available information. ETUs in the model were located in the capital city of the prefecture, and the respective health care workers were considered to be residing in the same city. Data on contact tracing and community safe burials were obtained from the Guinean Ministry of Health (GMoH) reports [7]; we do not have access to contact tracing forms at the level of individuals. The weekly number of new cases occurring in the general population were taken both from the WHO website (we consider both situation reports and patient database) and from GMoH reports [7]. The number of cases among health care workers were obtained from GMoH reports [7]. The data used are summarized in Fig. S2.

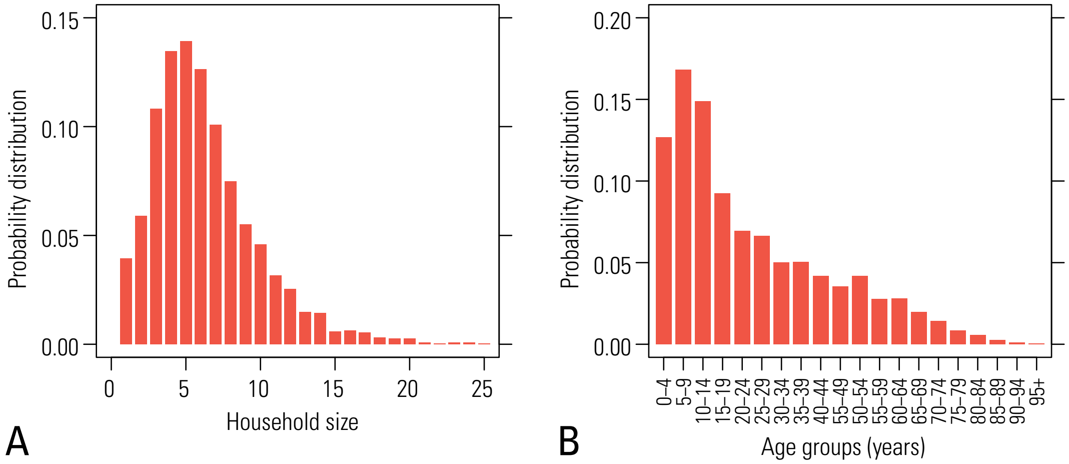

Figure S1: **A** Household size distribution of Guinea. **B** Age structure of Guinea.

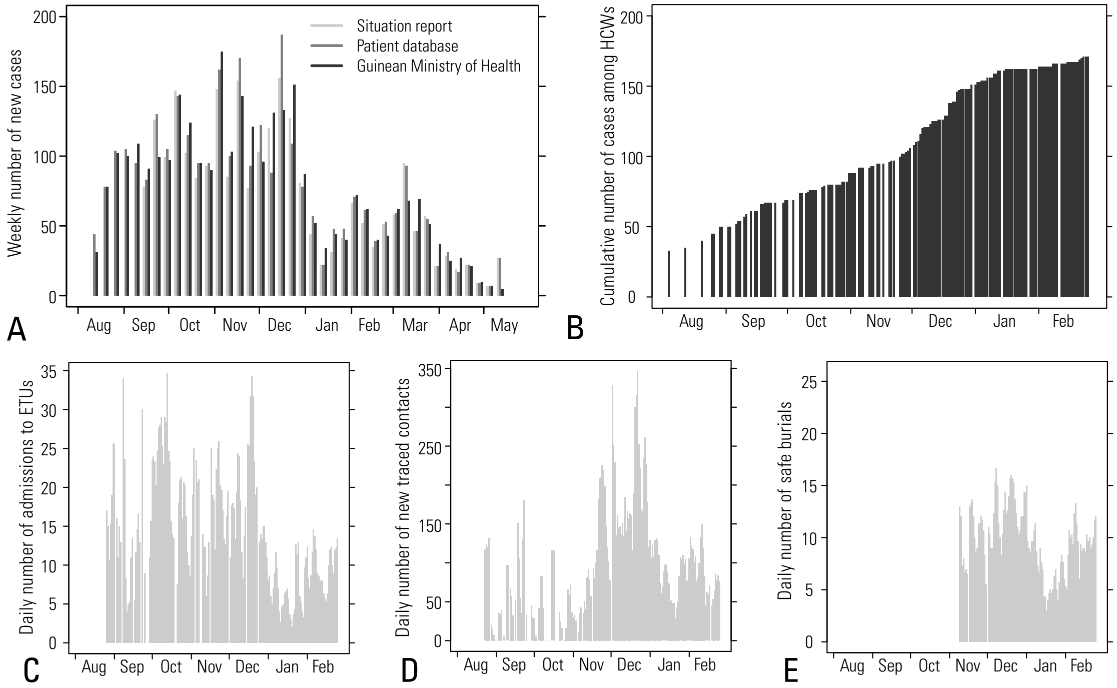

Figure S2: **A** Weekly number of new cases according to the WHO situation reports (light grey), the WHO patient database (grey) and the GMoH (dark grey). **B** Cumulative number of cases among health care workers. **C** Daily admissions to ETUs. **D** Daily number of new traced contacts. **E** Daily number of safe burials. The source for all data in **B**-**E** is the GMoH.

2 Transmission model and Ebola natural history

Each individual of the population, explicitly simulated as an agent of the individual-based model, has an associated epidemiological status. The different stages of Ebola transmission were modeled following Legrand et al. [8]: susceptible individuals can acquire infection after contact with an infectious individual and become exposed (asymptomatic). At the end of the incubation period (which we assumed equal to the latent period, as there is no evidence of pre-symptomatic Ebola transmission), exposed individuals become infectious (symptomatic). Infectious individuals can transmit infection at home (to both household members and members of the extended family). Infectious individuals at home then may either be hospitalized with 80% probability (see Sec. Results of the main text), die or recover. Hospitalized individuals may either die or recover. However, after recovery, a hospitalized individual remains in the hospital (though no longer infectious) for an additional period before being discharged. Deceased individuals may transmit infection during their funeral (to household members and to the extended family) and are then removed. Individuals belonging to the contact tracing pool are assumed to be properly checked daily and admitted to a hospital/ETU right at the onset of symptoms. Each (above mentioned) key time period used in the model for every individual is randomly sampled from an exponential distribution whose mean is reported in Tab. S1. This reflects observed heterogeneity in the disease history across individuals. The overall case fatality ratio is assumed to be 70.7% as reported in [9]. An extensive sensitivity analysis of the above parameters has already been performed in [10] for Liberia.

The model accounts for three routes of transmission: transmission in households and in the general

Table S1: Model parameters taken from the literature or directly inferred from the data.

Parameter	Value	Reference
Mean duration of incubation period	11.4 days	[9]
Mean time from symptom onset to death	7.5 days	[9]
Mean time from symptom onset to recovery for survivors	7.9 days	[10]
Mean time from symptom onset to hospitalization	5 days	[9]
Fraction of hospitalized cases	80%	inferred*
Mean time from hospitalization to death	4.2 days	[9]
Mean time from hospitalization to recovery for survivors	4.6 days	[10]
Mean time from hospitalization to dismissal for survivors	11.8 days	[9]
Mean time from death to burial	2 days	[8]
Overall case fatality ratio	70.7%	[9]

* *inferred from the data reported to the Guinean Ministry of Health as the crude approximation of the number of cases admitted to ETUs divided by the total number of cases. Since ETUs were open and never worked at full capacity over the period in which data is available, ETU admission could be considered as a proxy for admission to health care facilities.*

community (to the extended family) when infected individuals are at home, non-hospitalized; transmission in hospitals; and transmission during funerals (to household and extended family members).

In addition, the model takes into account two important features of the 2014-15 Ebola epidemic in Guinea: an age-dependent risk of infection – most infections occur among adults (see Sec. 4), and the presence of superspreading events – few cases responsible for a large number of infections (see Sec. 5).

Simulations were initialized with 52 infected individuals geographically distributed in four prefectures (namely Gueckedou, Conakry, Macenta and Dabola) based on information on the first confirmed cases reported to the WHO. The national-level simulations were run until there were 495 cases, at which point the simulated date was set to August 4, 2014 (according to GMoH reports [7]). This procedure, previously used in [3, 10], synchronizes all simulations to start with the observed conditions on this date.

2.1 Transmission within households

At time t, a non-hospitalized infectious individual j is able to transmit EVD to all other members of his/her household with the following force of infection:

$$\lambda_j(t) = \frac{\nu_j \beta_f}{N_{f_j}(t)}$$

where β_f is the transmission rate in households (assumed to be the same for all households), $N_{f_j}(t)$ is the household size at time t (thus excluding deceased and hospitalized members), and ν_j is a scalar factor accounting for the heterogeneity in transmission among individuals - specifically, ν_j is sampled from a gamma distribution having scale 4.78 and shape 0.2 (see Sec. 5).

The decision to make the force of infection inside a household depend on the number of members is a common assumption [3, 10, 11] reflecting the fact that members of larger households may have less frequent contact with the sick. This is especially relevant for Ebola as very close contact (i.e., mainly contact with body fluids) is required for transmission.

2.2 Transmission in the extended family

Each household is linked to six additional households representing its extended family, which are sampled with 50% probability in the same village and with 50% probability among households within 10 km to

reflect contacts residing in the same general community. In [12] it has been estimated that 11.5 traced contacts per case corresponds to about 30% detection probability of contact tracing investigation, which implies a total of 40 individuals at risk of infection (corresponding in the model to members of household and extended family). This information has been used to set the number of households composing the network of extended family to 6 (as the average household size in Guinea is about 6 individuals). Let α_j be the set of additional households (i.e., belonging to the extended family) for an infectious (non-hospitalized) individual j. Individual j transmits the infection to the members of α_j with force of infection

$$\lambda_j(t) = \frac{\nu_j \sigma \beta_f}{N_{\alpha_j}(t)}$$

where $N_{\alpha_j}(t)$ is number of individuals in α_j at time t and σ is the reduction of the transmission rate in the extended family ($0 \leq \sigma \leq 1$).

A sensitivity analysis assuming different number of additional households and different distance at which they are chosen was presented in [10] for the case of Liberia.

2.3 Transmission during burial ceremonies

Since it is likely that the bodies of those who died from EVD are still infectious, burial teams were progressively trained and equipped throughout the country to prevent disease spread driven by traditional funeral practices. In the model we assumed that funerals for individuals who died in ETUs were always conducted safely. In the beginning of the epidemic, all individuals who died both in general hospitals and at home (without being hospitalized) were assumed to be buried unsafely; after the beginning of November 2014 (as derived from the GMoH situation reports [7]), all who died in general hospitals were buried safely, while deceased at home had a daily probability of receiving a safe burial. In case of unsafe burial, the deceased individual j transmits EVD to his/her household members and members of the extended family α_j similarly to transmission in households, namely:

$$\lambda_j(t) = \frac{\nu_j \beta_b}{N_{f_j}(t)}$$

to household members and

$$\lambda_j(t) = \frac{\nu_j \sigma \beta_b}{N_{\alpha_j}(t)}$$

to members of the additional households, where β_b is the transmission rate in unsafe burials. Note that the same set of households involved in the general community transmission is assumed also for burial ceremonies.

In particular, we assume that individuals at risk during a burial ceremony are those belonging to the household and community settings (i.e., household members and members of the six additional households constituting the extended family of the deceased). This follows the idea that the specific network of contacts who are at highest risk of contracting the disease from a symptomatic Ebola case is almost the same as those who would be involved in the preparation of the body for the burial ceremony (for a description of traditional burial procedures see [13]).

2.4 Transmission in hospitals and ETUs

The limited availability of dedicated facilities over the territory of Guinea, especially in the first months of the epidemic, caused Ebola patients to be admitted also to general hospitals, where specific protocols for EVD management were not easily applied. As widely reported by official agencies [7, 14], a large number of health care workers had contracted the disease while caring for Ebola patients, and many have died. Many infections occurred in the period from October to December 2014 in non-Ebola health

care centers, probably as a consequence of the limited supply of personal protective equipment in these facilities [15]; the poor operating conditions of hospitals allowed the spread of infection not only to HCW but also to other patients. Thus it is reasonable to assume, as we did in our model, that non-Ebola patients in hospitals were also at risk of contracting the disease. In particular, we assumed that an Ebola case hospitalized to a non-dedicated facility was able to transmit infection to HCW and to other patients, for instance through contacts in rooms and waiting areas.

This scenario is detailed as follows. Individuals infected with Ebola have an 80% probability of being hospitalized. At every time step of the simulation, a symptomatic Ebola case seeking care is assigned to an ETU in the same prefecture where he/she lives, provided that an ETU with unoccupied beds exists in that prefecture; otherwise he/she gets hospitalized for one day into the closest hospital with unoccupied beds; if there is no such hospital, the Ebola case is not hospitalized. The following day the patient in hospital is either assigned to the closest ETU with unoccupied beds, or remains in the same hospital. Afterwards, the same procedure is used to hospitalize non-Ebola cases, who are randomly sampled from the population until full capacity of all hospitals in Guinea is reached. Non-Ebola cases remain hospitalized for 7 days on average [10].

The choice of explicitly modeling hospitals requires an additional free parameter regulating the transmission within this setting. However, the availability of data on recorded cases among HCW and the inclusion of such data in the likelihood function allows the estimation of this additional parameter though, obviously, with some uncertainty.

An Ebola-infectious individual j, admitted to a general hospital, transmits the infection to both susceptible hospitalized individuals and to health care workers with force of infection

$$\lambda_j(t) = \frac{\nu_j \beta_h}{N_{h_j}(t)}$$

where β_h is the transmission rate in hospital, and $N_{h_j}(t)$ is the overall number of hospitalized individuals and HCW in hospital h_j.

An infectious individual j, hospitalized in ETU, transmits the infection only to HCW, with force of infection equal to the force of infection in general hospitals with the transmission rate in hospital, β_h, rescaled by a 0.05 factor, to account for the lower infection probability of HCW in this setting with respect to general hospitals, consistent with the assumptions from previous modeling studies [16, 10].

2.5 Overall force of infection and probability of being infected

At any time t of the simulation (we consider a time step $\Delta t = 1$ day), A susceptible individual i has a probability $p_i(t) = \delta(a_i)\left[1 - \exp\left(-\Delta t \sum_j \lambda_j(t)\right)\right]$ of being infected from each infectious individual j in the population, where a_i is the age of individual i, and $\delta(a_i)$ is the age-dependent risk of infection: $\delta(a_i) = 1$ for the most affected age group (i.e., individuals aged 15 and over) and $\delta(a_i) = \delta = 0.246$ for individuals aged 0-14 (as estimated in Sec. 4).

The transmission rates across different settings β_f, β_h, β_b and the scaling factor σ depend on the specific population model used and on socio-demographic factors, and thus need to be estimated.

2.6 Modeling non-pharmaceutical intervention measures

We consider three non-pharmaceutical intervention measures that were performed during the outbreak: i) operation of ETUs; ii) community safe burials; iii) contact tracing investigation.

2.6.1 ETU

We introduced ETUs in our model whose opening dates, locations, number of beds and number of HCWs reflect the existing situation, as derived from publicly available information such as The Humanitarian

Data Exchange (`https://data.hdx.rwlabs.org`), local newspapers, NGO reports. We assume that there is no transmission between patients in ETUs, and transmission to HCW is reduced by 95% compared with hospitals (i.e., the transmission rate in ETUs is $0.05\beta_h$). In sum, hospitalizing cases in ETUs has three beneficial effects: 1) the period during which a case can transmit infection is shortened (since he can transmit in the community only before hospitalization); 2) deceased cases are always safely buried and thus do not transmit infection during funerals; 3) hospitalization of the case may trigger a contact tracing investigation.

2.6.2 Community safe burial

The GMoH reports the daily number of total burials and of safe burials in the community [7]. We computed the daily probability $B(t)$ of being safely buried in the community as the ratio between safe and total burials. For each day of the simulation t, every individual deceased at that time in the community (i.e., who was not hospitalized) had a probability $B(t)$ of being buried safely, thus preventing transmission events during funerals ($\beta_b = 0$).

2.6.3 Contact tracing investigation

The GMoH reports the daily number of contacts followed in contact tracing investigation [7], while the number of new contacts followed each day is not available; indeed, the daily number of new contacts listed for contact tracing is available, but this does not correspond to the number of contacts that were actually followed due to poor compliance of the population (resistance etc.). Thus we compute a proxy for the number of new contacts followed per case at day t, $F(t)$, as the ratio between the total number of followed contacts over the period $[t, t+21)$ and the total number of cases over the same period. This quantity suffers from two limitations: first, the denominator is the total number of cases instead of the total number of cases admitted to hospital/ETU (both triggering contact tracing investigation) since admission data are available for ETUs only; however, since we estimate the hospitalization rate in ETUs to be $\approx 83\%$, the number of cases is a good proxy for the number of cases admitted either to an ETU or to hospital. Second, we consider a period of 21 days because every contact was followed for that period, but this procedure would imply a nearly constant number of new daily cases; however, the recorded maximum number of weekly cases over the entire epidemic course was about 160, thus variation in the number of cases over a 21-day period is not remarkable. The obtained indicator for the contact tracing is significantly negative correlated to the number of observed cases (see discussion in the main text and Fig. S3). We analyze whether this correlation holds also for alternative definitions of F. Specifically, we tried by integrating over 7, 14, and 28 days period (instead of 21 days used as baseline). In all cases we found a significant negative correlation ($p < 0.001$) between our indicator of the contact tracing and the daily number of observed cases.

In [12] it has been estimated that the probability of identifying a case through contact tracing depends linearly on the number of contacts followed; in particular, for instance, 11.5 traced contacts per case correspond to about 30% detection probability. We use this information to derive from $F(t)$ the daily probability of following a contact, $\Phi(t)$. Therefore in our model, at each time step, for each individual admitted either to hospital or to ETU, we sampled from a Bernoulli distribution of probability $\Phi(t)$ to determine whether his household was followed or not. The same procedure was applied to each additional household in his extended family network. Once an individual belonging to a household followed by contact tracing became symptomatic, he was hospitalized on the same day of symptom onset (provided that beds were available).

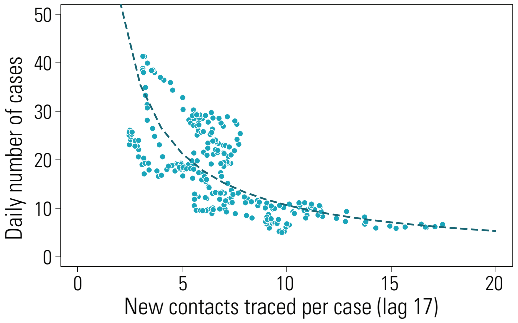

Figure S3: Scatter plot of observed daily number of cases and daily number of traced contacts as determined by equation $F(t)$. Note that the same indicator of contact tracing is used also in the main text.

3 Markov chain Monte Carlo calibration

Unlike [10], where the data points used for model calibration referred to an epidemic phase characterized by an almost exponential growth rate because interventions were not very intensive, the dataset used for model calibration in this study refers to a period where intensive interventions were ongoing. In particular, each of the three interventions implemented mainly acts in reducing transmission in one specific setting: ETUs reduce transmission in health care settings; community safe burials reduce transmission at funerals; and contact tracing reduces transmission in households and extended family. Therefore, we estimated the posterior distribution of all four unknown parameters of the model (which are all related to Ebola transmissibility, namely β_h, β_b, β_f, σ); the parameter vector is denoted by Θ. The prior distributions of the four parameters are uniform in $[0, 1000]$ for the transmission rates and in $[0, 1]$ for σ. The posterior distribution of Θ was explored by Markov chain Monte Carlo (MCMC) sampling applied to the likelihood of the weekly number of cases among HCW (as reported to the Guinean Ministry of Health) and in the general population in the entire country of Guinea (as reported in the patient database of the WHO). Specifically, by assuming the number of cases among HCW and in the general population to be Poisson distributed around the mean and independent for each time interval, we can write the total likelihood as the product of two distinct likelihood functions, L_{hcw} and L_{gp}. In detail L_{hcw} is defined as $L_{hcw} = \prod_{i=1}^{n} P(w_i(\Theta); k_i)$, n is the number of data points, P is the probability of observing k_i events from a Poisson distribution with mean $w_i(\Theta)$, where k_i is the observed number of cases among HCW in time interval i, $w_i(\Theta)$ is the estimated mean (computed over 50 simulations) number of cases among HCW in time interval i from a candidate parameter vector Θ. We define L_{gp} analogously. Thus, no spatial information is used in the computation of the likelihood.

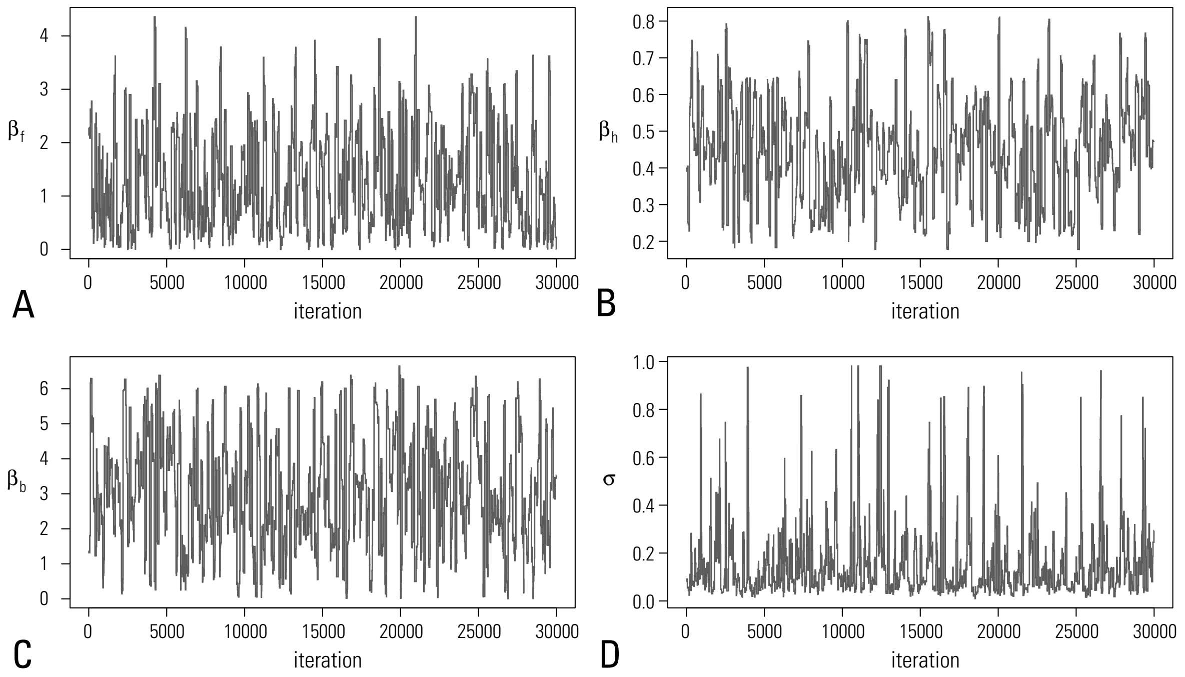

Figure S4: Estimated parameter values at each iteration of the MCMC algorithm.

Table S2: Estimates of transmission parameter.

Parameter	mean (95% CI)
Transmission rate (day^{-1}) in household, β_f	1.340 (0.048–3.424)
Transmission rate (day^{-1}) in hospital, β_h	0.445 (0.200–0.754)
Transmission rate (day^{-1}) at funeral, β_b	3.070 (0.269–6.121)
Scaling factor for extended family, σ	0.155 (0.027–0.702)

Random-walk Metropolis-Hastings sampling is used to estimate Θ. In particular, the chain is initialized by sampling each component of Θ from uniform distributions. Then, at each iteration, the likelihood of a new candidate vector of parameters is evaluated and the candidate is either accepted or rejected following the usual Metropolis-Hastings algorithm [17]. As simulations are stochastic, whenever new candidate vectors are not accepted 10 times in row, the likelihood of the current parameter set is re-evaluated and the new likelihood accepted with probability 1 [18]. This ensures that the chain does not remain trapped in a local maximum. The values of a new candidate parameter vector are randomly sampled from a normal distribution with mean equal to the current transmission rate and variance ϵ^2.

The four values of ϵ are chosen in such a way to guarantee a good acceptance rate. We perform 40,000 iterations and discard the first 10,000 iterations as a burn-in period. We check convergence by considering several different starting points and by visual inspection (see Fig. S4). Moreover, for each parameter we perform t-tests on its posterior distributions by sampling 100 random values over different subsets of iterations (namely, 10,000 vs 20,000, 10,000 vs 30,000, and 20,000 vs 30,000); we found that the obtained posterior distributions are not significantly different.

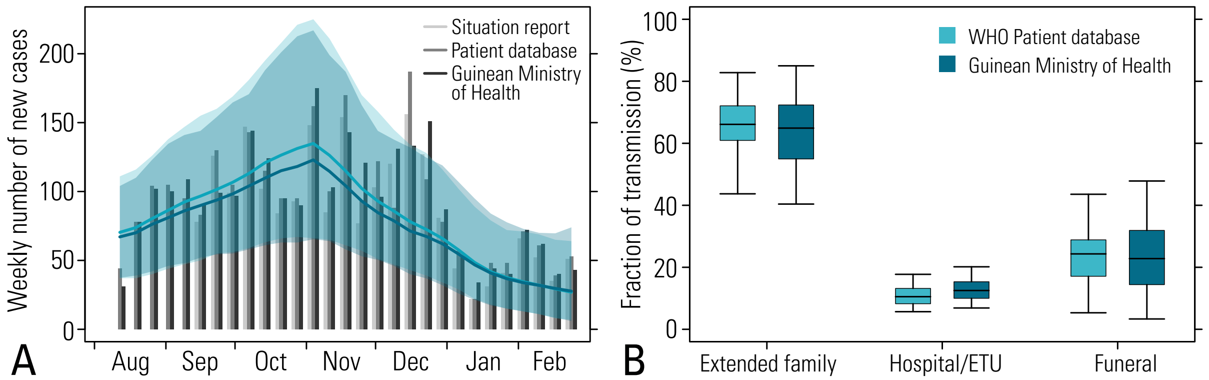

Figure S5: **A** Weekly number of cases as reported in the data (WHO situation report - light grey, WHO patient database - dark grey, Guinean Ministry of Health - darker grey) and estimated by the model using the weekly cases reported to the Guinean Ministry of Health in the likelihood (mean - blue lines, 95%CI - light blue area). **B** Estimated fraction of transmission by setting as of February 25, 2015, in the model where the weekly data reported in the WHO patient database (light blue) or the weekly data reported to the Guinean Ministry of Health (blue) are used in the likelihood.

The reported mean values and confidence intervals are computed by running an independent stochastic realization with the parameter vector of every tenth iteration of the 30,000 iterations of the MCMC algorithm. The resulting set of 3,000 realizations thus reflects both the stochasticity of the model and

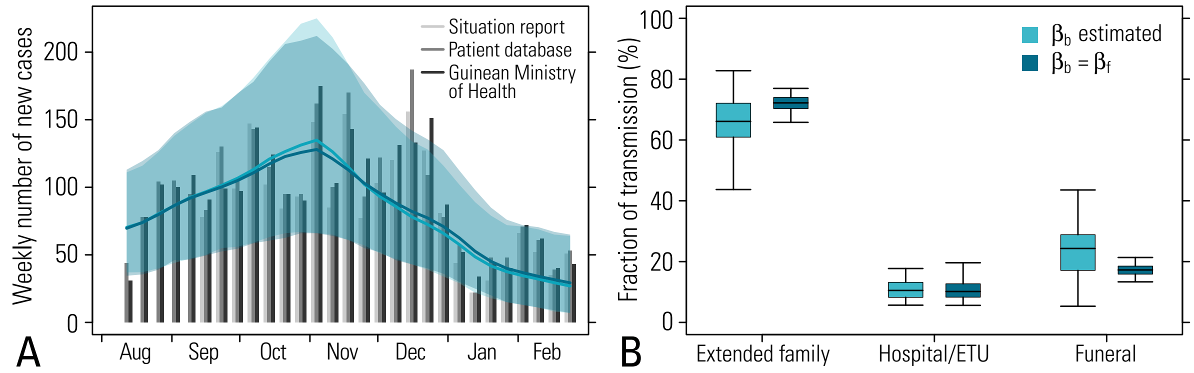

Figure S6: **A** Weekly number of cases as reported in the data (WHO situation report - light grey, WHO patient database - dark grey, Guinean Ministry of Health - darker grey) and estimated by the model where β_b is assumed equal to β_f (mean - blue lines, 95%CI - light blue area). **B** Estimated fraction of transmission by setting as of February 25, 2015, in the model where β_b is estimated (light blue) and where it is kept equal to the transmission rate in between household members (blue).

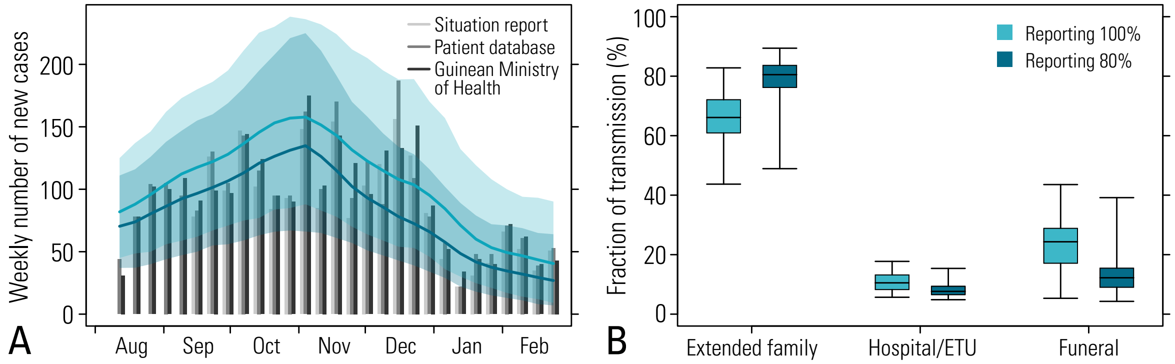

Figure S7: **A** Weekly number of cases as reported in the data (WHO situation report - light grey, WHO patient database - dark grey, Guinean Ministry of Health - darker grey) and estimated by the model assuming 80% reporting rate for cases in the overall population (mean - blue lines, 95%CI - light blue area) – for HCW we assume 100% reporting rate. **B** Estimated fraction of transmission by setting as of February 25, 2015, in the model assuming 80% reporting rate (light blue) and 100% reporting rate (blue).

the uncertainty in model parameters estimates (whose values are reported in Tab. S2).

In addition, we performed three sensitivity analyses on model calibration. First, in the definition of the likelihood, we substituted the number of weekly cases reported in the WHO Patient Database with the number of weekly cases reported to the Guinean Ministry of Health. As shown in Fig. S5, model estimates are consistent for these two alternative sources of data used in the definition of the likelihood. Second, as in [10], we also performed an analysis assuming $\beta_b = \beta_f$, thus reducing the number of parameters to be estimated through the MCMC procedure. As shown in Fig. S6, the model is still able to capture the weekly number of cases, and the estimated mean fraction of cases by setting is similar to the baseline scenario, although estimates are less variable in this new scenario with an additional model constraint (Fig. S6). Third, we assume that only 80% of Ebola cases have been reported to the WHO. The resulting estimated weekly number of cases follows the same temporal trend of the scenario assuming reporting rate 100% (see Fig. S7A). Moreover, also the estimated fraction of cases by setting remains quite consistent between the two scenarios (Fig. S7B).

4 Age-specific risk of infection

The number of Ebola cases reported to the WHO [14] is highly variable by age. As of February 22, 2015, the cumulative number of infections in individuals aged 15+ years is 2,557 (which is about 84% of infections recorded until that date), despite this age group representing only about the 54% of the population of Guinea. Such a peculiar pattern of the 2014–2015 Ebola epidemic could have been due to different behaviors followed by individuals of different ages; for instance, it is more likely that adults take care of sick individuals and thus have greater chance to come into contact with body fluids of infected Ebola cases. A first approximation of such a complex phenomenon can be modeled by assuming an age-specific risk of infection.

In order to estimate age-specific risk of infection, we developed a simple compartmental model. The

set of ordinary differential equations regulating the compartmental model is

$$\begin{aligned}
\dot{S}_1 &= -\delta S_1 \beta \sum_{j=1}^{2} \frac{I_j + Y_j}{N} \\
\dot{I}_1 &= \delta S_1 \beta \sum_{j=1}^{2} \frac{I_j + Y_j}{N} - 2\gamma I_1 \\
\dot{Y}_1 &= 2\gamma I_1 - 2\gamma Y_1 \\
\dot{R}_1 &= 2\gamma Y_1 \\
\dot{S}_2 &= -S_2 \beta \sum_{j=1}^{2} \frac{I_j + Y_j}{N} \\
\dot{I}_2 &= S_2 \beta \sum_{j=1}^{2} \frac{I_j + Y_j}{N} - 2\gamma I_2 \\
\dot{Y}_2 &= 2\gamma I_2 - 2\gamma Y_2 \\
\dot{R}_2 &= 2\gamma Y_2
\end{aligned}$$

where

- S_j is the number of susceptible individuals belonging to age group j (age group 1 corresponds to individuals ≤ 15 years old; age group 2 corresponds to individuals 15+ years old);
- I_j is the number of individuals belonging to age group j who are in the first infectious class. We assume two classes for infectious individuals (I and Y) in such a way that the resulting generation time is Gamma-distributed;
- Y_j is the number of individuals belonging to age group j who are in the second infectious class;
- R_j is the number of recovered individuals belonging to age group j;
- δ is the relative risk of infection of younger individuals (< 15 years old) with respect to adults (15+ years old);
- β is the transmission rate;
- γ is the rate of loss of infectiousness.
- $N = N_1 + N_2$ is the population size where $N_1 = 4,676,171$ and $N_2 = 5,381,804$ are the sizes of the two age groups respectively.

The model has two free parameters, namely the transmission rate β and the relative risk of infection of younger individuals. Parameters were estimated by exploring with MCMC the binomial likelihood of the number of cases in the two different age groups up to October 10, 2014 (i.e. before incidence flattens/decreases, possibly as a consequence of improved interventions) as reported in the WHO Situation Reports [14]. This allows us to estimate the basic reproduction number observed in the early epidemic phase.

Specifically, the likelihood was defined as:

$$L = \prod_{t=1}^{6} \prod_{j=1}^{2} B(c_j(t), N_j, p_j(t))$$

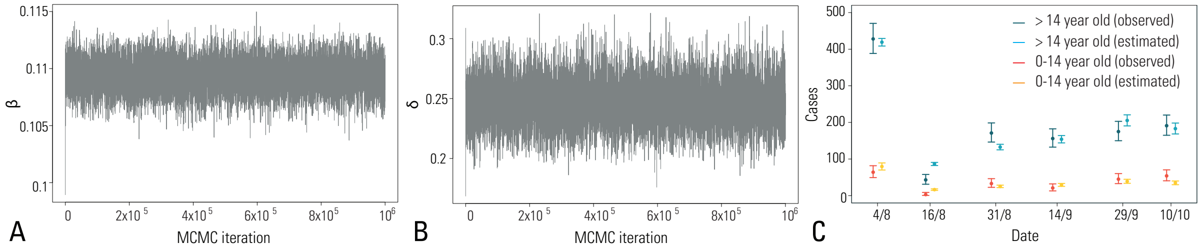

Figure S8: **A** Value of the transmission rate of the compartmental model for different iterations of the MCMC algorithm. **B** Value of the relative risk of infection of the compartmental model for different iterations of the MCMC algorithm. **C** Number of cases over time for children 0–14 years old and for individuals 15+ years old as predicted by the compartmental model and as reported in the WHO situation reports.

where index $t = 1, \ldots, 6$ represents time steps, $j = 1, 2$ represents age groups, $B(k, n, p)$ is the probability mass function of a binomial distribution (i.e. the probability of observing k successes in n experiments, each of which yields success with probability p), $c_j(t)$ is the number of cases observed at time t in age group j, N_j is the size of age group j, and $p_j(t)$ is the fraction of new cases at time t in age group j as estimated by the model.

A Metropolis-Hasting algorithm (1,000,000 iterations) with normal proposal distributions was used. We check convergence by considering several different starting points and by visual inspection. Results are reported in Fig S8. β was estimated to be 0.114 days^{-1} (95%CI: 0.111-0.117), δ was estimated to be 0.247 (95%CI: 0.212-0.283).

The basic reproduction number of the model is $R_0 = \frac{\beta}{\gamma}(\delta\frac{N_1}{N} + \frac{N_2}{N})$, as resulting from the next generation matrix approach introduced in [19]. This yields to an estimated R_0=1.176 (95%CI: 1.169–1.184).

5 Heterogeneous transmissibility

A characteristic feature of the current Ebola epidemic in Guinea is the presence of heterogeneity in transmissibility[20, 21], i.e., a small fraction of infected individuals is responsible for the large majority of secondary cases. We analyzed data on the transmission chain of 152 infections in Guinea [21] by fitting them with a negative binomial distribution (see Fig. S9). We found that the maximum likelihood distribution has dispersion parameter k=0.20 (95%CI: 0.13-0.31), which is in very good agreement with the result reported in [20].

Following the argument presented in [22], in our transmission model, which follows the classical Poisson approach for transmission events, we modeled the negative binomial distribution as a Poisson distribution where the mean transmission rate $\hat{\beta}$ is multiplied by a gamma distribution with shape 0.2 (i.e., the dispersion parameter k of the estimated negative binomial distribution) and scale 4.78 (i.e., $(1-p)/p$, where p is the probability of the estimated negative distribution, which can be computed as $k/(k+\mu)$).

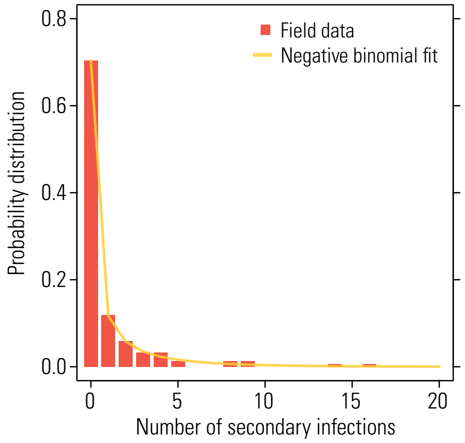

Figure S9: Probability distribution of the number of secondary cases as observed in [21] (red rectangles) and from fitting a negative binomial distribution (orange line).

6 Reproduction number over time

To estimate the reproduction number of the model over time we assumed the the number of daily cases $C(t)$ as estimated by the model at time t can be approximated by a Poisson process:

$$C(t) \approx Pois(R_t \sum_{s=1}^{t} T_g(s)C(t-s))$$

where T_g is the generation time distribution of the model (approximated by a Gamma distribution of parameters shape 2.39 (SE: 0.014) and rate 0.13 (SE: 0.0008), see [10]), and R_t is the instantaneous reproduction number at time t. The likelihood is therefore

$$L = \prod_{t=1}^{T} P(C(t), R_t \sum_{s=1}^{t} T_g(s)C(t-s))$$

where here $P(k, \lambda)$ is the probability mass function of a Poisson distributions (i.e. the probability of observing k events if these events occur with a known rate λ). For each simulated epidemic obtained by running the calibrated model, R_t were estimated by maximum likelihood. Mean and 95% credible intervals of R_t were obtained by analyzing the entire Markov chain.

7 Geographical spread

The geographical spread of the epidemic at the regional level is depicted in Fig. S10. All datapoints for all regions, except Boke, fall inside the 95%CI of model predictions. The predicted trend in the cumulative number of cases differs from those reported in the data for Labe, Mamou, and Boke regions, which had observed a total of 7, 17, and 47 cases by February 25, 2015, respectively. Moreover, Mamou and Boke data showed a fluctuating cumulative number of reported cases probably attributable to mis- and/or under- reporting of cases. Together with the non-availability of complete information on the performed interventions at a sub-national scale, this is the second reason why the model is calibrated on national level data only.

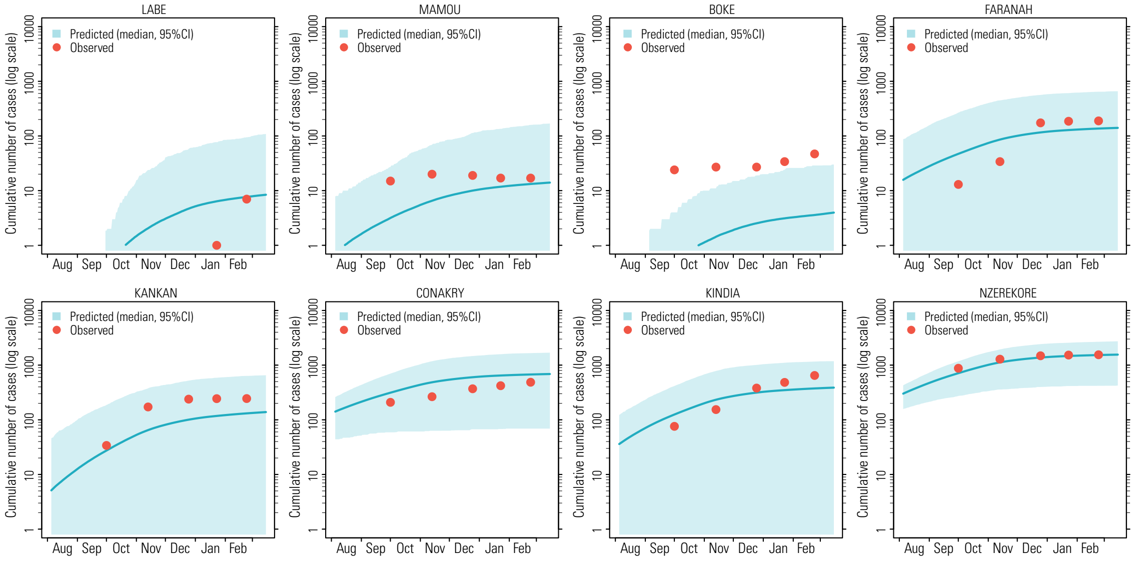

Figure S10: Cumulative number of cases (on the log10 scale) in the 8 Guinean regions as predicted by the model (line: mean, shaded area: 95%CI) and as reported to the Guinean Ministry of Health (points).

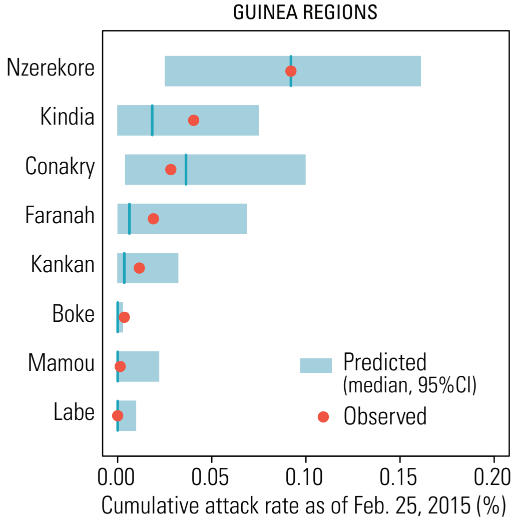

Figure S11: Cumulative attack rate (expressed in percentage) as of February 25, 2015 in the 8 Guinean regions as predicted by the model (line: mean, shaded area: 95%CI) and as reported to the Guinean Ministry of Health (points).

The analysis of cumulative attack rates confirms the results based on cumulative number of cases (compare Fig. S11 with Fig. 1B in the main text). This is due to the relative homogeneity of the population size of Guinea regions (ranging from about 0.7 million individuals in Mamou, up to 2 million

individuals in Kankan).

8 Analysis of performed intervention measures

As expected, the cross-correlation between ETU admissions and Ebola cases data shows a highly significant maximum at lag 0 days (Fig. S12A), while the community safe burial probability is not significantly correlated with the number of reported cases for any lag, with the exception of a low correlation at very long lags (i.e., over 35 days – see Fig. S12B) that has no clear biological interpretation.

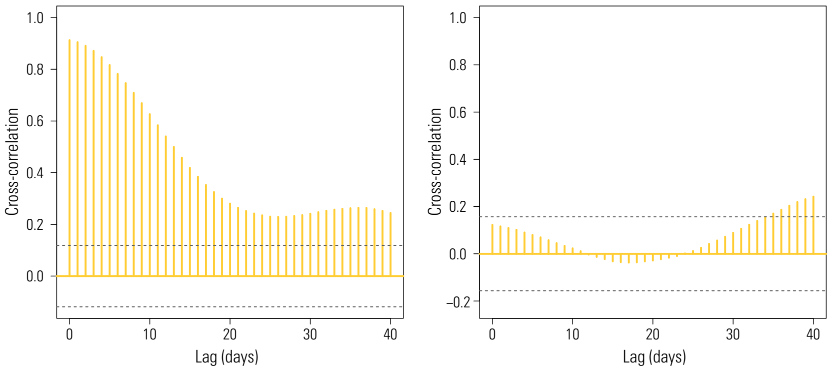

Figure S12: **A** Cross-correlation between the average number of cases admitted to ETU and the average number of reported cases. **A** Cross-correlation between the average probability of being safely buried in the community and the average number of reported cases. Bars exceeding the blue lines represent statistically significant lags. All data used in this analysis are reported by the Guinean Ministry of Health; averages are computed on a moving window of 15 days, i.e., one week previous and one week following the data point) over time.

9 Probability of disease elimination

The cumulative number of cases depending on the number of traced contacts per case, corresponding to Fig. 5A in the main text, is shown in Fig. S13. In S14 we perform a sensitivity analysis on the vaccine efficacy by considering VE=75% and 100%. We use the setting of a randomized trial with delayed rings and with vaccine administered only to individuals 18+ years old.

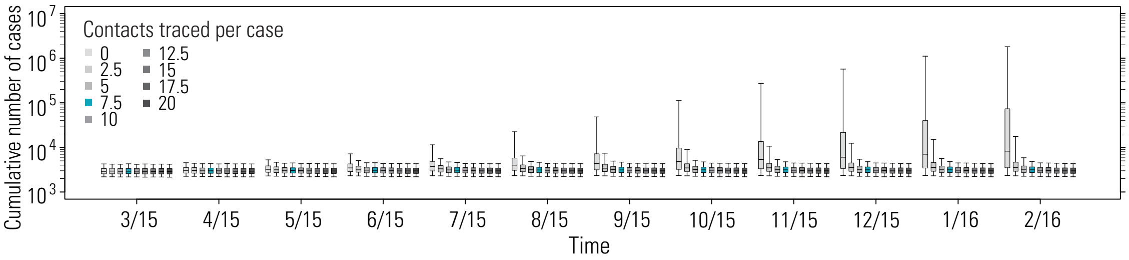

Figure S13: Boxplot for the cumulative number of cases over time assuming different values for the number of traced contacts per case (same scenarios as in Fig. 5A of the main text). Blue boxplots represent a situation comparable to what was observed in April 2015. The number of traced contacts match the data until February 25, 2015; then it is assumed to be constant over time until the end of the simulation, at the level reported in the legend.

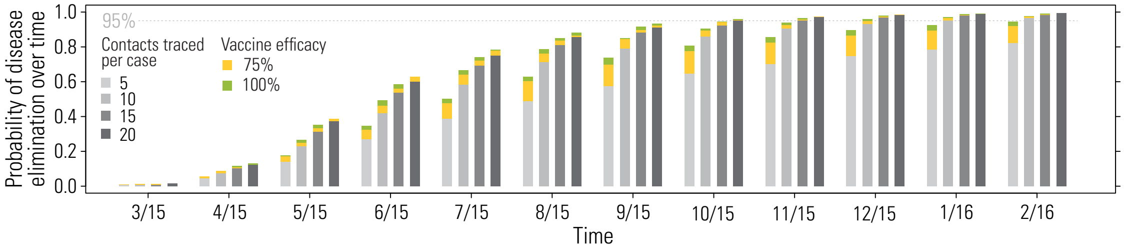

Figure S14: Increase in the probability of EVD elimination in Guinea for vaccine efficacy of 100% and 75% and different baseline of contact traced per case. When vaccine efficacy is 75%, the impact of vaccination on the probability of EVD elimination is most noticeable at low levels of contact tracing. The number of traced contacts match the data until February 25, 2015; then it is assumed to be constant over time until the end of the simulation, at the level reported in the legend.

References

1. Institut National de la Statistique Guinée (2014) Résultats préliminaires du Troisième Recensement Général et de la Population et de l'Habitation. (in French).
2. Merler S, Ajelli M (2010) The role of population heterogeneity and human mobility in the spread of pandemic influenza. *Proc R Soc B* 277(1681):557–565.
3. Merler S, Ajelli M, Pugliese A, Ferguson NM (2011) Determinants of the Spatiotemporal Dynamics of the 2009 H1N1 Pandemic in Europe: Implications for Real-Time Modelling. *PLOS Comput Biol* 7(9):e1002205.
4. The DHS Program (2007) Demographic and Health Survey 2007.
5. Institut National de la Statistique Guinée (2007) Guinée - Annuaire des statistiques sanitaires 2007. (in French).
6. WHO Regional Office for Africa (2010) Country Health Profile - Guinea Factsheets of Health Statistics 2010. (in French).
7. Guinean Ministry of Health (2014-2015) Rapport de la Situation Epidemiologique. (in French).
8. Legrand J, Grais RF, Boélle PY, Valleron AJ, Flahault A (2007) Understanding the dynamics of Ebola epidemics. *Epidemiol Infect* 135(04):610–621.
9. WHO Ebola Response Team (2014) Ebola Virus Disease in West Africa — The First 9 Months of the Epidemic and Forward Projections. *N Eng J Med* 371(16):1481–95.
10. Merler S et al. (2015) Spatiotemporal spread of the 2014 outbreak of Ebola virus disease in Liberia and the effectiveness of non-pharmaceutical interventions: a computational modelling analysis. *Lancet Infect Dis* 15(2):204–211.
11. Ferguson NM et al. (2006) Strategies for mitigating an influenza pandemic. *Nature* 442(7101):448–452.
12. Ajelli M et al. (2015) The 2014 Ebola virus disease outbreak in Pujehun, Sierra Leone: epidemiology and impact of interventions. *BMC Med* 13:281.
13. Hewlett BS, Amola RP (2003) Cultural contexts of Ebola in northern Uganda. *Emerg Infect Dis* 9(10):1242–8.
14. WHO Global Alert and Response (2014-2015) Situation reports: Ebola response roadmap.
15. WHO Global Alert and Response (2015) One year into the Ebola epidemic.
16. Meltzer MI et al. (2014) Estimating the Future Number of Cases in the Ebola Epidemic – Liberia and Sierra Leone, 2014–2015. *MMWR Morb Mortal Wkly Rep* 63:1–14.
17. Gilks WR (2005) *Markov Chain Monte Carlo.* (Wiley Online Library).
18. Cauchemez S, Valleron AJ, Boëlle PY, Flahault A, Ferguson NM (2008) Estimating the impact of school closure on influenza transmission from Sentinel data. *Nature* 452(7188):750–754.
19. Diekmann O, Heesterbeek JAP, Metz JAJ (1990) On the definition and the computation of the basic reproduction ratio R0 in models for infectious diseases in heterogeneous populations. *J Math Biol* 28(4):365–382.
20. Althaus CL (2015) Ebola superspreading. *Lancet Infect Dis* 15(5):507–508.

21. Faye O et al. (2015) Chains of transmission and control of Ebola virus disease in Conakry, Guinea, in 2014: an observational study. *Lancet Infect Dis* 15(3):320–6.

22. Lloyd-Smith JO, Schreiber SJ, Kopp PE, Getz WM (2005) Superspreading and the effect of individual variation on disease emergence. *Nature* 438(7066):355–359.
